# Ovarian Hyperandrogenism and Response to Gonadotropin-releasing Hormone Analogues in Primary Severe Insulin Resistance

**DOI:** 10.1210/clinem/dgab275

**Published:** 2021-04-26

**Authors:** Isabel Huang-Doran, Alexandra B Kinzer, Mercedes Jimenez-Linan, Kerrie Thackray, Julie Harris, Claire L Adams, Marc de Kerdanet, Anna Stears, Stephen O’Rahilly, David B Savage, Phillip Gorden, Rebecca J Brown, Robert K Semple

**Affiliations:** 1 University of Cambridge Metabolic Research Laboratories, Wellcome Trust-MRC Institute of Metabolic Science, Addenbrooke’s Hospital, Cambridge, UK; 2 National Institute for Health Research Cambridge Biomedical Research Centre, Cambridge, UK; 3 Diabetes, Endocrinology, and Obesity Branch, National Institute of Diabetes and Digestive and Kidney Diseases (NIDDK), National Institutes of Health (NIH), Bethesda, MD, USA; 4 Histopathology Department, Cambridge University Hospitals NHS Foundation Trust, Cambridge, UK; 5 Pediatric Endocrinology Unit, University Hospital, Rennes, France; 6 National Severe Insulin Resistance Service, Wolfson Diabetes & Endocrine Clinic, Addenbrooke’s Hospital, Cambridge University Hospitals NHS Foundation Trust, Cambridge, UK; 7 Centre for Cardiovascular Science, Queen’s Medical Research Institute, University of Edinburgh, Edinburgh, UK

**Keywords:** Polycystic ovary syndrome, insulin receptor, hyperinsulinemia, lipodystrophy, androgen, GnRH analogue

## Abstract

**Context:**

Insulin resistance (IR) is associated with polycystic ovaries and hyperandrogenism, but underpinning mechanisms are poorly understood and therapeutic options are limited.

**Objective:**

To characterize hyperandrogenemia and ovarian pathology in primary severe IR (SIR), using IR of defined molecular etiology to interrogate disease mechanism. To extend evaluation of gonadotropin-releasing hormone (GnRH) analogue therapy in SIR.

**Methods:**

Retrospective case note review in 2 SIR national referral centers. Female patients with SIR with documented serum total testosterone (TT) concentration.

**Results:**

Among 185 patients with lipodystrophy, 65 with primary insulin signaling disorders, and 29 with idiopathic SIR, serum TT ranged from undetectable to 1562 ng/dL (54.2 nmol/L; median 40.3 ng/dL [1.40 nmol/L]; n = 279) and free testosterone (FT) from undetectable to 18.0 ng/dL (0.625 nmol/L; median 0.705 ng/dL [0.0244 nmol/L]; n = 233). Higher TT but not FT in the insulin signaling subgroup was attributable to higher serum sex hormone–binding globulin (SHBG) concentration. Insulin correlated positively with SHBG in the insulin signaling subgroup, but negatively in lipodystrophy. In 8/9 patients with available ovarian tissue, histology was consistent with polycystic ovary syndrome (PCOS). In 6/6 patients treated with GnRH analogue therapy, gonadotropin suppression improved hyperandrogenic symptoms and reduced serum TT irrespective of SIR etiology.

**Conclusion:**

SIR causes severe hyperandrogenemia and PCOS-like ovarian changes whether due to proximal insulin signaling or adipose development defects. A distinct relationship between IR and FT between the groups is mediated by SHBG. GnRH analogues are beneficial in a range of SIR subphenotypes.

Polycystic ovary syndrome (PCOS) affects 5% to 20% of women and is a major cause of morbidity and subfertility. As captured in various diagnostic criteria, PCOS is characterized by clinical or biochemical evidence of androgen excess, chronic ovulatory dysfunction (manifesting as menstrual irregularity), and polycystic ovarian morphology, typically demonstrated sonographically by an increased number of peripheral preantral-like follicles (1).

The association of PCOS with diabetes was first recognized a century ago (2), and in recent decades the association between insulin resistance (IR) and hyperandrogenism has been widely reported (3). From the 1970s onwards, severe hyperandrogenism was observed in particularly extreme forms of IR, including those due to genetic insulin receptor deficiency and anti-insulin receptor autoantibodies (4-6), and those associated with lipodystrophy (7-9). Before onset of diabetes, IR is associated with compensatory hyperinsulinemia, which can be extreme. The effects of excessive insulin action on the ovary, which appear to be independent of the insulin receptor (INSR), are suggested to drive ovarian hyperandrogenism in common PCOS (10).

A systematic assessment of ovarian pathology in severe insulin resistance (SIR) has not been conducted, although scattered reports have described massive, bilateral ovarian enlargement (11, 12) and ovarian neoplasia (13) associated with severe genetic insulin receptor dysfunction (Donohue syndrome). We now describe the spectrum of hyperandrogenemia and ovarian pathology in a large cohort of patients with SIR, including many with defined monogenic or autoimmune etiologies. We show that primary IR, whether caused by proximal defects in insulin signaling or lipodystrophy, is sufficient to cause ovarian hyperandrogenism with ovarian morphology indistinguishable from common PCOS. IR and sex hormone–binding globulin (SHBG) concentration show a distinct positive relationship in the insulin signaling subgroup, consistent with a direct role for liver insulin signaling in suppressing hepatic SHBG production in other forms of IR. Finally, we evaluate the efficacy of GnRH analogue therapy in lowering testosterone levels and improving androgenic symptoms in women with SIR, extending prior evidence that pulsatile gonadotropins play a permissive role in IR-associated ovarian hyperandrogenism.

## Patients and Methods

### Patients

We performed a retrospective review of case records from 2 national referral centers for SIR at the National Institutes of Health, USA (since 1977), and University of Cambridge, UK (since 1992). All patients were referred based on clinical and/or biochemical evidence of SIR and/or lipodystrophy and were studied using procedures approved by the institutional review board of the National Institute of Diabetes and Digestive and Kidney Diseases or by the UK Research Ethics Committee. Each participant or their legal guardian provided written, informed consent, and minors provided verbal or written consent in accordance with local regulations. Studies were conducted in accordance with the principles of the Declaration of Helsinki.

### Inclusion Criteria

Patients (all female) met 1 or more of the following criteria: (1) body mass index (BMI) <30 kg/m^2^ with normal glucose tolerance and fasting hyperinsulinemia >150 pmol/L, and/or peak insulin >1 500 pmol/L after a 75 g oral glucose challenge; (2) BMI <30kg/m^2^ and diabetes with insulin requirements >3 units/kg/day; (3) a disorder known to cause SIR (eg, lipodystrophy). All patients were evaluated by an endocrinologist and appropriate endocrine testing was performed to exclude secondary causes of IR and/or lipodystrophy where clinical suspicion was raised. Dysthyroidism and hyperprolactinemia were excluded prior to referral. Similarly, where indicated, adrenal sources of hyperandrogenemia were excluded by the reviewing endocrinologist. Patients of all ages were included in this study; however, given the focus on the interaction between hyperinsulinemia and the hypothalamic–pituitary–gonadal (HPG) axis, some analyses were restricted to life stages during which the HPG axis is likely to be active. Pubertal status was assessed formally in a subset of patients (Tanner breast stage II-IV categorized as “midpubertal”; Tanner breast stage V or postmenarche categorized as “postpubertal”). If clinical assessment of pubertal status was not documented, we pragmatically used a threshold of 10 years and above to define the group with likely HPG axis activation. In a further subset of patients, menopausal status was determined from chart review (ie, menopause documented in chart, age >60 years, or luteinizing hormone (LH)/follicle-stimulating hormone (FSH) in the menopausal range).

### Biochemical Assays

All patients underwent biochemical evaluation as part of clinical care. Analyses, including serum total testosterone (TT), sex hormone–binding globulin (SHBG) and fasting insulin, were performed in accredited clinical diagnostic laboratories of a range of referring hospitals, using various platforms over a 40-year period. The majority of TT levels were determined by immunoassay, with a subset of around 10% to 20% measured by mass spectrometry. For all individuals, results reported as below or above the assay range were treated as being at the lower or upper limit of the assay range, respectively, for the purposes of graphical representation and statistical analysis. In patients with a documented SHBG level, free testosterone (FT) was estimated using the Vermeulen method, assuming an albumin concentration of 45 g/L.(14)

### Insulin Resistance Subphenotyping

Causal genetic mutations, where reported, were identified by Sanger sequencing or exome/genome sequencing as part of clinical care or programs of research into the genetic basis of SIR. Type B IR was diagnosed by the presence of anti-insulin receptor autoantibody detected by immunoprecipitation, as previously described (15). Patients were classified into 3 groups based on clinical, biochemical and genetic evaluation: (1) lipodystrophy, either generalized or partial (including congenital and acquired syndromes); (2) primary disorders of insulin signaling, due to either (a) loss-of-function mutations in *INSR* (encoding the insulin receptor), *PIK3R1* (encoding the p85α regulatory subunit of PI3-kinase), *AKT2* (encoding protein kinase B), or *TBC1D4* (encoding AS160), or (b) type B IR; or (3) SIR without lipodystrophy of unknown but suspected genetic etiology (“idiopathic SIR”).

### Ovarian Pathology

Ovarian tissue samples were obtained as part of clinical care, processed in accredited histopathology laboratories and reported by local histopathologists. Available hematoxylin and eosin-stained sections were further reviewed by an independent gynecological histopathologist blinded to clinical presentation and diagnosis but not to the age of the patient.

### Statistics

Summary statistics presented are median and interquartile range (IQR), unless indicated otherwise. TT, FT, fasting insulin or SHBG levels were compared among multiple groups using a 2-tailed, nonparametric Kruskal–Wallis test followed by post hoc pairwise Wilcoxon rank sum test with Bonferroni correction. Association between fasting insulin and testosterone or SHBG was assessed using Spearman’s test. Statistical significance was declared at *P* < .05. All analyses were performed in R version 3.5.2 (2018-12-20).

## Results

### Prevalence and Severity of Hyperandrogenemia in Primary Severe Insulin Resistance

**Figure 1.**
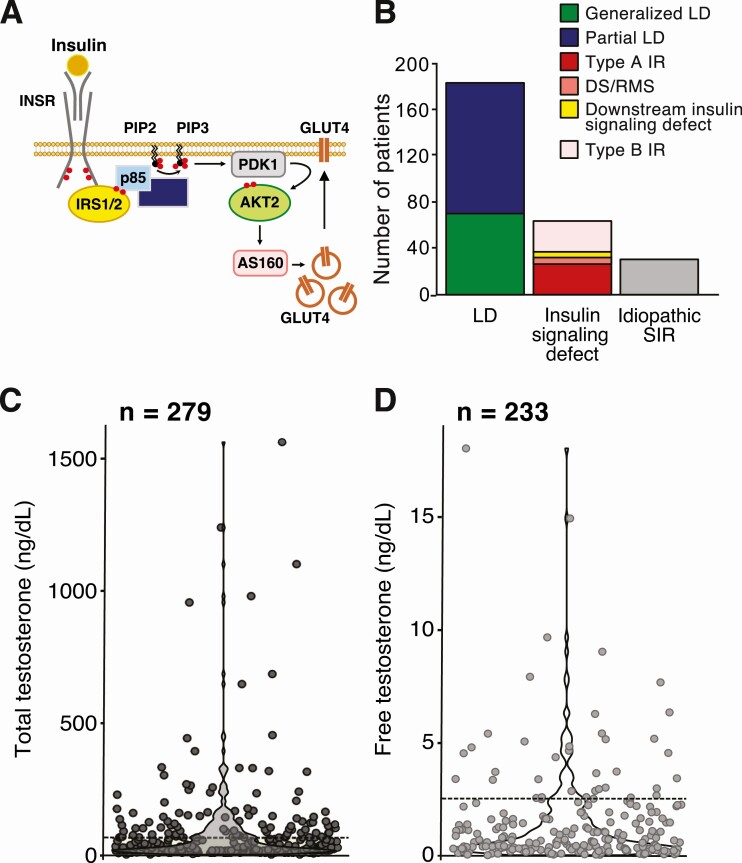
Total and free serum testosterone levels in 279 patients with primary syndromes of severe insulin resistance. (A) Intracellular signaling cascade mediating insulin-stimulated glucose uptake. Binding of insulin to the insulin receptor (INSR) activates its intrinsic kinase activity, leading to INSR autophosphorylation and tyrosine phosphorylation of insulin receptor substrates (IRS) 1 and 2 (phosphate groups shown in red). The p85 α regulatory subunit of PI3-kinase interacts with phosphorylated IRS proteins, activating and approximating the p110 catalytic subunit to the plasma membrane, resulting in appearance of the phospholipid second messenger phosphatidylinositol (3-5)-trisphosphate (PIP3). This in turn acts as a docking site for a range of effector proteins including Protein Kinase B (AKT), activated by PIP3-dependent phosphorylation by phosphoinositide-dependent kinase 1 (PDK1). One target of AKT2 in adipose tissue and skeletal muscle is the AKT substrate of 160 kDa (AS160, encoded by the *TBC1D4* gene), phosphorylation of which leads to translocation of GLUT4 glucose transporters to the plasma membrane. Loss-of-function mutations in *INSR*, *PIK3R1* (encoding the p85ɑ subunit of PI3K), *AKT2* and *TBC1D4* (encoding AS160) have all been associated with monogenic insulin resistance. (B) Composition of severe insulin resistance subgroups. The lipodystrophy group included patients with generalized or partial lipodystrophy (genetic or acquired). The insulin signaling defect group included patients with pathogenic variants in the insulin receptor (Donohue syndrome (DS), Rabson–Mendenhall syndrome (RMS) as well as less severe “type A” insulin resistance), pathogenic variants in genes encoding downstream insulin signaling components (p85 α, AKT2, and AS160) and insulin receptor dysfunction due to acquired autoantibodies (“type B” insulin resistance). The idiopathic severe insulin resistance group consisted of a highly selected group of patients with severe insulin resistance of presumed monogenic etiology but no known genetic defects and no insulin receptor autoantibodies. (C) Total serum testosterone levels in female patients aged 4 months to 70 years with a range of genetic and acquired syndromes of insulin resistance, measured in various referring hospitals over a 40 year period (n = 279). Total testosterone of 70 ng/dL, approximating the upper limit of normal in most clinical immunoassays, is indicated for reference. (D) Calculated free testosterone levels in 233 patients who had a documented sex hormone–binding globulin (SHBG) concentration. Upper limit of normal (2.4 ng/dL) indicated for reference.

A total of 279 patients (aged between 4 months and 70 years) with confirmed or likely primary SIR had a serum TT level available for review. Of these, 185 (66%) had partial or generalized lipodystrophy (114 and 71 patients, respectively) and 65 (23%) had a primary disorder of insulin signaling, affecting the insulin receptor or components of the downstream insulin signaling cascade (Fig. 1A and 1B). These included 9 patients with severe biallelic variants causing Donohue or Rabson–Mendenhall syndrome, while the remaining 24 had less extreme IR manifesting peripubertally, caused by monoallelic *INSR* pathogenic variants (historically known as “type A” IR). Four patients had IR due to pathogenic variants in *PIK3R1*, encoding the p85α regulatory subunit of PI3-kinase (reported in (16)), 1 had a pathogenic variant in downstream protein kinase B (*AKT2*, reported in (17)), and 1 had a pathogenic variant in *TBC1D4,* encoding AKT substrate of 160 kDa (AS160), which is implicated in insulin-stimulated GLUT4 translocation (18, 19). A further 29 patients (10%) had SIR without lipodystrophy, insulin receptor autoantibodies, or a confirmed genetic diagnosis, but suspected to be of monogenic etiology (“idiopathic SIR”). Of 152 patients in the cohort whose medication records were readily available, the proportions taking metformin, insulin, or other oral hypoglycemic agents were 50%, 48% and 20%, respectively. Only 8% were on a hormonal contraceptive preparation, and 2% were taking spironolactone.

Serum TT concentrations across the study cohort, measured using different assays in multiple laboratories, ranged from undetectably low to 1562 ng/dL (54.2 nmol/L) (Fig. 1C). The median testosterone level was 40.3 ng/dL (1.4 nmol/L; IQR 20-94.5 ng/dL or 0.694-3.279 nmol/L). One-third of patients (94/279, 34%) had a testosterone level above 70 ng/dL (2.4 nmol/L), which approximates the upper limit of normal in adult females in most clinical immunoassays. Thirty-six of 279 (13%) had a serum testosterone in excess of 150 ng/dL (~5 nmol/L), approximately twice the upper limit of normal. Among 233 patients with a documented SHBG level, calculated serum FT concentration ranged from undetectably low to 18.0 ng/dL (0.625 nmol/L) (Fig. 1D). The median FT level was 0.705 ng/dL (0.0244 nmol/L; IQR 0.340-1.781 ng/dL or 0.0118-0.0618 nmol/L). Forty of 233 (17%) had a calculated FT level above the upper limit of normal (2.4 ng/dL or 0.083 nmol/L) (Fig. 1D).

### Severity of Hyperandrogenemia in Different Insulin Resistance Subphenotypes

**Figure 2.**
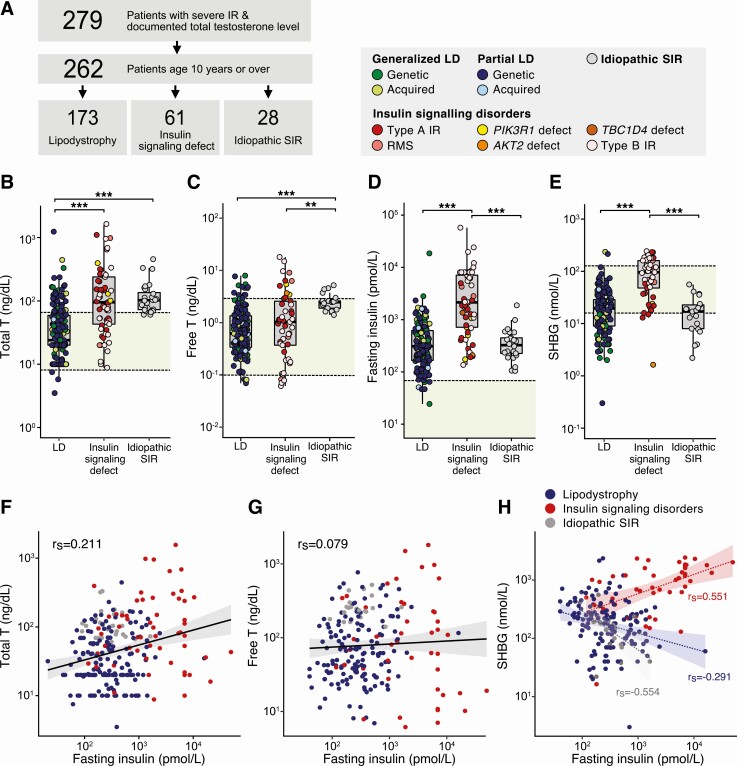
Severity of hyperandrogenemia in different insulin resistance subphenotypes. (A) Selection of 262 individuals with primary severe insulin resistance for subphenotype analyses. 17 patients under 10 years were presumed to have an inactive hypothalamus-pituitary-gonadal axis and thus excluded from the analysis. The remaining patients were divided into 3 clinical subgroups (lipodystrophy (LD), insulin signaling defect, idiopathic severe insulin resistance). (B-E) Total serum testosterone, calculated free serum testosterone, fasting insulin and sex hormone–binding globulin (SHBG) by severe insulin resistance subgroup. Symbol color denotes clinical/genetic subphenotypes (see legend). Subgroups were compared using the Kruskal-Wallis test followed by pairwise Wilcoxon rank sum test with Bonferroni correction (***P < .005, **P < .01). Population references ranges indicated (green shading). (F-H) Association between fasting insulin and total testosterone (F), free testosterone (G), and SHBG (H), colored by clinical subgroup. Spearman’s rank correlation coefficient (r_s_) values in (H) are subgroup specific. Abbreviations: IR, insulin resistance; LD, lipodystrophy; NS, not significant; RMS, Rabson–Mendenhall syndrome; SIR, severe insulin resistance.

Seventeen patients were under 10 years old and therefore deemed unlikely to have a pubertal HPG axis. TT and FT, SHBG, and fasting insulin were evaluated in the remaining 262 patients aged 10 or above, grouped by clinical subphenotype (Fig. 2A and Table 1). Within this cohort, median LH and FSH concentrations were 5.0 U/L (IQR 2.5-9.7) and 5.0 U/L (IQR 3.3-9.7), respectively. Median LH/FSH ratio was 0.84 (IQR 0.54-1.3) and did not differ significantly between patients with lipodystrophy and those with defects in proximal insulin signaling (data not shown). Among 173 individuals with lipodystrophy, TT ranged from below the assay limit of detectability to 1239 ng/dL (43.0 nmol/L) (median 24.0 ng/dL, IQR 20.0-59.0 ng/dL) while FT ranged from below detectability to 7.93 ng/dL (0.275 nmol/L) (median 0.619 ng/dL, IQR 0.338-1.35 ng/dL). TT and FT levels in generalized lipodystrophy (whether genetic, acquired and autoimmune, or of unknown cause) were no different from those in partial lipodystrophy (genetic, acquired, or idiopathic). Moreover no significant differences were observed in TT or FT concentrations among genetic subgroups in generalized or partial lipodystrophy. Among 61 patients with defects in insulin signaling, TT ranged from below detectability to 1561 ng/dL (54.1 nmol/L) (median 95.1 ng/dL, IQR 43.0-239 ng/dL) and FT ranged from below detectability to 18.0 ng/dL (0.613 nmol/L) (median 1.03 ng/dL, IQR 0.37-2.57 ng/dL). No significant differences were observed in TT or FT levels among patients with pathogenic variants in insulin receptor or proximal insulin signaling components, or type B IR.

**Table 1.** Serum testosterone levels in syndromes of primary severe insulin resistance (age 10 years or above)

Syndrome	n	Age, years	Total testosterone, ng/dL	Free testosterone, ng/dL	Insulin, pmol/L	SHBG, nmol/L
**1) Lipodystrophy**	**173**	**27 (17-42)**	**24.0 (20.0-59.0)**	**0.61 (0.34-1.35)**	**267 (143-533)**	**17 (8-22.8)**
a. Generalized lipodystrophy	60	17 (14-24)	23.0 (10.0-42.7)	0.66 (0.35-1.21)	374 (204-736)	12 (6-20)
i. Genetic	45	17 (14-22)	24.0 (10.0-48.0)	0.72 (0.34-1.46)	285 (163-683)	9 (5.1-17.8)
*AGPAT2*	30	17 (14-22)	23.5 (10.0-52.8)	0.73 (0.34-1.52)	315 (168-746)	8.0 (5-15)
*BSCL2*	5	14 (13-16)	30.0 (29.0-40.9)	0.77 (0.63-1.31)	606 (161-682)	38.5 (28.8-43.3)
*LMNA*	3	17 (14-18)	10.0 (10.0-34.0)	0.36 (0.31-1.28)	262 (204-321)	15 (23-36.5)
*Unknown*	7	21 (20-24)	14.4 (10.0-41.0)	0.63 (0.41-0.84)	252 (195-590)	17 (12.5-21.5)
ii. Acquired	15	24 (17-30)	21.0 (18.5-34.0)	0.62 (0.43-0.91)	513 (354-854)	13 (12-23)
b. Partial lipodystrophy	113	37 (21-47)	24.4 (20.0-63.4)	0.60 (0.34-1.39)	238 (116-358)	23 (23-12.9)
i. Genetic	105	37 (23-47)	24.4 (20.0-63.5)	0.57 (0.34-1.45)	217 (116-335)	23 (12-38)
* LMNA*	49	49 (38-25)	28.1 (20.0-56.0)	0.55 (0.38-1.32)	207 (116-307)	23 (15-36.5)
* PPARG*	13	32 (25-48)	20.0 (10.0-41.6)	0.38 (0.23-0.99)	336 (138-730)	22.5 (10.25-33.3)
* PYCT1A*	1	10	3.5	0.08	390	18
* Unknown*	42	39 (23-47)	26.7 (20.0-84.5)	0.65 (0.32-1.78)	212 (123-315)	24 (12.4-39.5)
ii. Acquired	8	25 (14-43)	24.0 (20.0-43.5)	0.60 (0.40-0.98)	614 (284-750)	24.5 (19.3-33.8)
**2) Insulin signaling disorder**	**61**	**21 (15-40)**	**95.1 (43.0-239)**	**1.03 (0.37-2.57)**	**1844 (617-6111)**	**96 (48-160)**
a. Genetic insulin signaling defect	35	16 (14-22)	115 (62.1-225)	1.58 (0.80-3.50)	1172 (409-1937)	43 (21.8-102.8)
*INSR (RMS)*	5	16 (13-16)	80.7 (49.0-648)	1.32 (0.93-2.65)	2430 (1521-3687)	108 (68.5-160)
*INSR (Type A IR)*	24	18 (14-24)	106 (53.2-171)	0.62 (0.53-6.29)	1235 (376-1890)	34.5 (20.25-39.5)
*PIK3R1*	4	13 (13, 14)	186 (122-280)	4.43 (3.94-4.92)	525 (362-693)	62.5 (43.3-81.8)
*AKT2*	1	41	95.1	3.7	180	1.6
*TBC1D4*	1	14	249	N/A	1172	N/A
b. Type B insulin resistance	26	42 (25-51)	61.8 (23.7-216)	0.49 (0.17-1.17)	6528 (1885-6944)	120 (93-179)
**3) Idiopathic severe insulin resistance**	**28**	**27 (18-31)**	**102 (72.8-135)**	**2.47 (1.89-2.61)**	**280 (194-387)**	**17 (8-22.8)**
**Total**	**262**	**26 (42-17)**	**40.3 (20.0-94.5)**	**0.75 (0.35-1.93)**	**335 (166-875)**	**22.9 (11.9-48.3)**

Median (interquartile range) values presented. Summary data for Severe Insulin Resistance subphenotypes are presented in bold font.

Abbreviations: IR, insulin resistance; N/A; data unavailable; RMS, Rabson–Mendenhall syndrome; SHBG, sex hormone binding globulin.

TT was significantly higher in the insulin signaling group and idiopathic SIR group than the lipodystrophy group (Kruskal–Wallis test followed by pairwise Wilcoxon rank sum test with Bonferroni correction, *P* < .005) (Fig. 2B). Patients with insulin signaling disorders did not have significantly different TT levels from those with idiopathic SIR (Fig. 2B). In contrast, patients in the idiopathic SIR group had significantly higher FT than both the lipodystrophy and insulin signaling groups (Fig. 2C), while there was no significant difference in FT between the lipodystrophy and insulin signaling groups.

### Association Between Hyperinsulinemia, Sex Hormone–binding Globulin and Hyperandrogenemia in Primary Severe Insulin Resistance

To assess whether differences in TT and FT concentrations between clinical subgroups are related to degree of hyperinsulinemia and SHBG levels, we compared fasting insulin and SHBG, where available, in patients aged >10 years with lipodystrophy, insulin signaling defects or idiopathic SIR. Fasting insulin concentration ranged from 21 pmol/L to over 49 000 pmol/L (n = 245, median 335 pmol/L, IQR 166-875 pmol/L; Table 1), and was above the upper limit of normal (60 pmol/L) in 97% of all patients. In those on insulin analogue therapy, or with impaired beta-call function, fasting insulin concentration is likely to have underestimated the severity of IR, while metformin use will have reduced resistance. Fasting insulin was significantly higher in patients with insulin signaling defects than in those with lipodystrophy or idiopathic SIR (Fig. 2D). A comparable observation was made using homeostatic model assessment of IR (HOMA-IR) in lieu of fasting insulin, although this model is not validated in monogenic IR (data not shown).

Patients with insulin signaling disorders also had significantly higher SHBG than other SIR subtypes (Fig. 2E). Among all patients age >10 years, SHBG ranged from <1 to 243 nmol/L (n = 220, median 22.9 nmol/L, IQR 11.9-48.2 nmol/L; normal range 18-144 nmol/L in nonpregnant, adult females). No alternative cause of perturbed serum SHBG (eg, cirrhosis) was identified in any patient. Among patients with lipodystrophy, 45% (67/150) and 1.3% (2/150) had an SHBG level below or above the normal range, respectively; these proportions were 8% (4/49) and 27% (13/49) among patients with insulin signaling disorders, and 52% (11/21) and 0% (0/21) among patients with idiopathic SIR (Fig. 2E). Fasting insulin and TT were positively associated (Spearman’s rank correlation coefficient r_s_ = 0.211, *P* = 9.07 × 10^–04^; Fig. 2F), whereas a positive association was not seen between fasting insulin and FT (r_s_ = 0.079, *P* = .251; Fig. 2G). Amongst patients with lipodystrophy and idiopathic SIR, fasting insulin and SHBG were negatively associated (r_s_ = –0.291, *P* = 3.58 × 10^–04^ and r_s_ = –0.554, *P* = .0113, respectively), whereas a positive association between these 2 variables was seen among patients with insulin signaling disorders (r_s_ = 0.551, *P* = 7.21 × 10^–05^) (Fig. 2H), potentially explaining this discrepancy.

### Hypothalamic–Pituitary–Gonadal Axis Activity and Hyperandrogenemia in Severe Insulin Resistance

**Figure 3.**
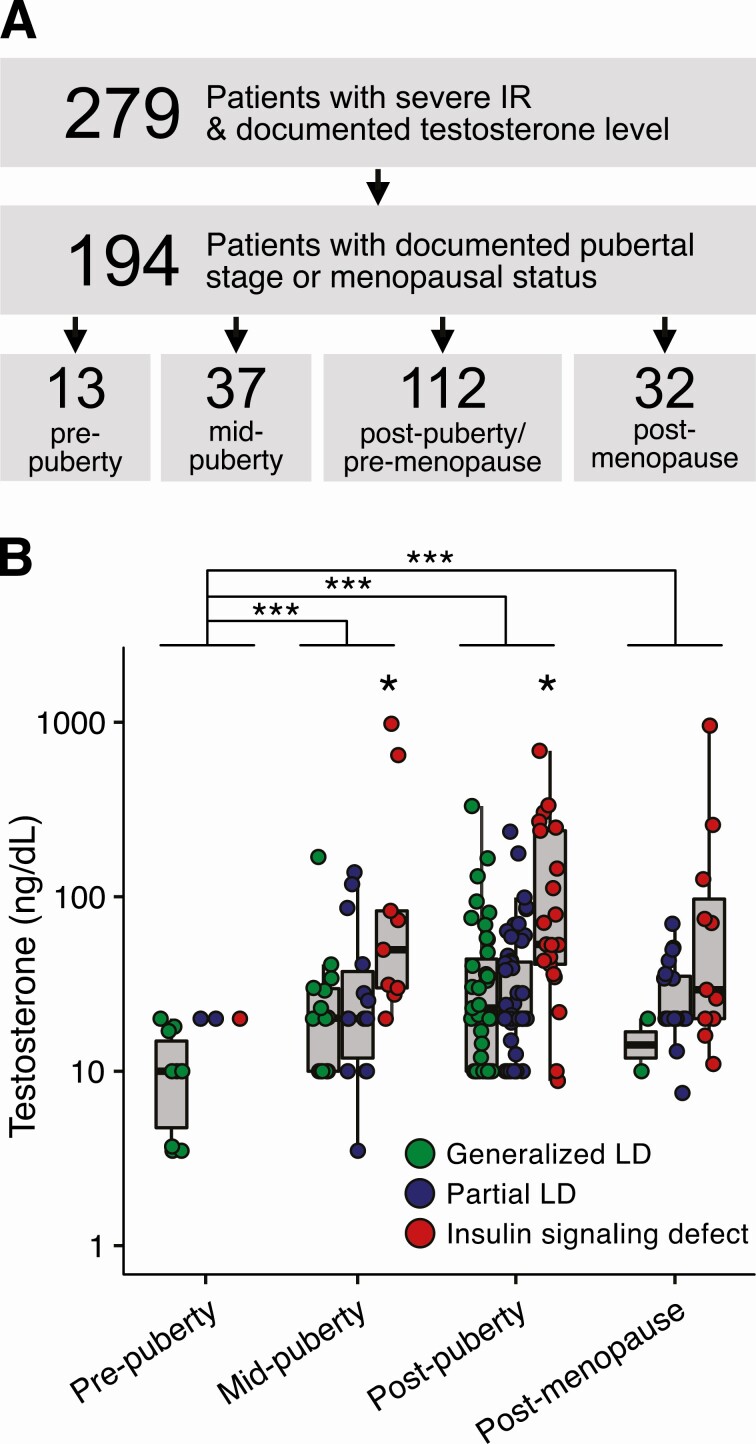
Hypothalamic–pituitary–gonadal (HPG) axis activity and hyperandrogenemia in severe insulin resistance. (A) Selection of individuals with primary severe insulin resistance for subgroup analyses. For 194 patients, hypothalamic-pituitary-gonadal axis (HPG) status was documented in, or inferred from, their clinical charts. Individuals were categorized as prepuberty, midpuberty, postpuberty or postmenopause. Each group was further subdivided into 3 clinical subgroups (generalized LD, partial LD, insulin signaling defect). (B) Total serum testosterone by HPG axis activity and clinical subgroup. Testosterone levels in patients with different HPG status were compared using the Kruskal-Wallis test followed by pairwise Wilcoxon rank sum test with Bonferroni correction (***P < .005). Within each HPG status subgroup, patients with lipodystrophy and insulin signaling disorders were compared using the Wilcoxon rank sum test (*P < .05). Abbreviations: IR, insulin resistance; LD, lipodystrophy.

To determine the extent to which androgen excess in primary SIR is affected by HPG axis activity, we analyzed 194 patients of all ages whose pubertal/menopausal status was documented in clinical records. They included 65 patients with generalized lipodystrophy, 87 with partial lipodystrophy and 42 with defective insulin signaling, of whom 16 had a genetic *INSR* pathogenic variant, 25 had type B IR and 1 had a *TBC1D4* defect. Individuals were classified as prepubertal (n = 13), midpubertal (n = 37), postpubertal (n = 112), and postmenopausal (n = 32) (Table 2 and, Fig. 3A). None of the 13 prepubertal individuals (ie, lacking clinical evidence of HPG activation) had an elevated TT concentration (Fig. 3B), and as a group they had significantly lower TT levels compared to all other groups (Kruskal–Wallis test followed by pairwise Wilcoxon rank sum test with Bonferroni correction, *P* < .005) (Fig. 3B). No differences were observed between midpubertal or postpubertal patients, nor was lower TT observed after menopause (Fig. 3B). Within the midpubertal and post-pubertal subgroups, patients with insulin signaling disorders had significantly higher TT levels than patients with lipodystrophy (Wilcoxon rank sum test, *P* < .05), as observed in Fig. 2D, but no statistically significant difference was observed in the postmenopausal group (Fig. 3B).

**Table 2.** Interaction between hyperandrogenemia and hypothalamic–pituitary–gonadal axis activation in primary severe insulin resistance

Syndrome	n	Age, years	Total testosterone, ng/dL
**Prepuberty**	**13**	**7 (7-10)**	**10.0 (10.0-20.0)**
Generalized lipodystrophy	10	7 (7)	10.0 (5.3-15.2)
Partial lipodystrophy	2	10 (9.5-10.5)	20.0 (20.0-20.0)
Insulin signaling disorder	1	13	20.0
**Midpuberty**	**37**	**13 (11-15)**	**25.4 (20.0-41.0)**
Generalized lipodystrophy	14	13 (11-14)	20.2 (10.0-29.8)
Partial lipodystrophy	14	14 (12-15)	20.0 (12.5-37.8)
Insulin signaling disorder	9	12 (10-15)	49.7 (30.0-83.0)
**Postpuberty**	**112**	**27 (18-39)**	**24.2** (**20.0-57.3**)
Generalized lipodystrophy	39	20 (16-23)	23.0 (10.0-44.1)
Partial lipodystrophy	52	35 (27-43)	20.0 (20.0-42.3)
Insulin signaling disorder	21	25 (18-39)	52.3 (41.0-239)
**Postmenopause**	**32**	**53 (49-58)**	**20.0** (**20.0-44.7**)
Generalized lipodystrophy	2	45 (38-52)	15.0 (12.5-17.5)
Partial lipodystrophy	19	53 (47-57)	20.0 (20.0-35.0)
Insulin signaling disorder	11	52 (51-57)	29.3 (20.0-100)
**Total**	**194**	**27 (42-15)**	**20.8 (49.7-18.3)**

Median (interquartile range) values presented. Summary data for HPG status are presented in bold font.

### Ovarian Pathology Associated With Severe Insulin Resistance

**Figure 4.**
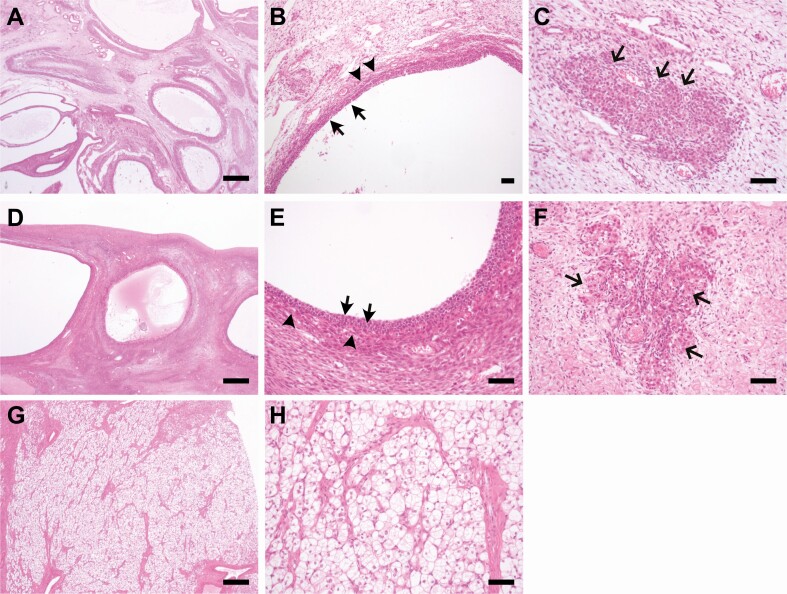
Histological appearances of the ovary in primary severe insulin resistance**. (A-C)** Patient 1 (Donohue syndrome) underwent bilateral oophorectomy aged 4 months after presenting with abdominal distention and respiratory distress. (A,B) Multiple follicular cysts lined by several layers of granulosa and theca cells (arrows and arrowheads, respectively). (C) Nests of eosinophilic and vacuolated luteinized cells within the ovarian stroma (arrows), in keeping with stromal hyperthecosis. Primordial follicles were present. Appearances consistent with PCOS. (D-H) Patient 7 (familial partial lipodystrophy, unknown genetic cause) presented with secondary amenorrhea and hyperandrogenism in her twenties, following which a left ovarian mass was identified. (D,E) Multiple follicular cysts in the left ovary lined by several layers of granulosa and theca cells (arrows and arrowheads, respectively). (F) Nests of eosinophilic and vacuolated luteinized cells within the ovarian stroma in keeping with stromal hyperthecosis (arrows). Appearances in keeping with PCOS. (G,H) Stromal cells with abundant vacuolated cytoplasm and small, round, centrally-located nuclei within the stroma, consistent with a steroid-secreting tumor. No significant cytological atypia or mitotic activity. Scale bars: 1 mm (A, D, G), 100 um (B, C, E, F, H).

To assess ovarian morphology associated with hyperandrogenemia in primary SIR, we reviewed the case notes of 9 patients who underwent either oophorectomy or ovarian wedge resection as part of clinical care, and for whom ovarian tissue or histological reports were available. Clinical, biochemical, and histological data are presented in Table 3. Representative histological images, where available, are provided in Fig. 4. Of the 9 patients, 4 had a proven *INSR* variant, 4 had partial lipodystrophy (3 associated with known genetic variants), and 1 had a heterozygous pathogenic variant in *AKT2* (previously reported in 4, 17, 20-29). Patient 1 had Donohue syndrome and presented in infancy with gross abdominal distention causing respiratory distress due to massive, bilateral ovarian enlargement (20). The remaining 8 individuals developed features of hyperandrogenism (typically oligo/amenorrhea and hirsutism) during their teens or early twenties. Eight of the 9 patients had underlying ovarian histological features consistent with PCOS, including multiple follicular cysts and stromal hyperthecosis. Six patients had documented hyperandrogenemia. Of the 9 patients, 1 (P7) subsequently developed a left ovarian steroid cell tumor aged 27 (Fig. 4D-4H), another (P9) developed worsening virilization at age 48 associated with a Sertoli–Leydig cell tumor, and a third (P3) died aged 50 due to a rapidly progressive metastatic cancer of presumed ovarian origin. Additionally, 2 patients (P4, P5) were found to have ovarian cystadenomas in their late teens.

**Table 3.** Ovarian pathology associated with primary severe insulin resistance

Patient	Clinical and genetic diagnosis	Clinical presentation	Age^*a*^, years	Insulin^*b*^, pmol/L	TT^*b*^, ng/dL	Ovarian pathology	Ref.
P1	**Donohue syndrome** *INSR* p.Cys264Tyr/p.Thr488Pro (compound heterozygous)	In infancy with abdominal distention and respiratory distress. Also growth retardation, lipoatrophy, acanthosis nigricans, hirsutism, hypertrichosis, and clitoromegaly. Bilateral oophorectomy due to respiratory distress. Received recombinant human IGF1.	0.33	19446	127	Massive bilateral ovarian enlargement (left 103 g, right 230 g). Multiple follicular cysts with stromal hyperthecosis, consistent with PCOS (Fig 4A-C).	(20)
P2	**Digenic insulin resistance with partial lipodystrophy** *PPARG (*A469ΔAAAiT) fs.156(stop 157) */ PPP1R3A* (C1984ΔAG) fs.662(stop 668) digenic heterozygous (rs587776687, rs527638422)	Post-puberty with an abdominal mass associated with hirsutism, secondary amenorrhea, acanthosis nigricans. Large bilateral fallopian and ovarian cysts on pelvic ultrasound, prompting unilateral oophorectomy. Postoperatively, hirsutism responded to combination cyproterone acetate and ethinylestradiol.	14	276	N/A	Abdominal mass due to large fallopian and ovarian cysts. Multiple follicular cysts with stromal hyperthecosis, consistent with PCOS.(25)	(21, 29)
P3	**Insulin resistance with diabetes** *AKT2* p.Arg274His heterozygous (rs121434593)	At age 16 with secondary amenorrhea, hirsutism, acne, virilization, prompting bilateral ovarian wedge resection. Regular menses thereafter, and 2 successful pregnancies. At age 35 with acromegaloid facies and hands. Diabetes aged 38 with worsening hirsutism, necrobiosis lipoidica and high insulin requirements. Total abdominal hysterectomy and left salpingo-oophorectomy aged 42 for menorrhagia. At age 50 with large right sided pelvic mass with hydronephrosis, liver and pulmonary nodules. Presumed metastatic epithelial ovarian cancer. Died 7 days later.	16	N/A	N/A	Ovarian wedge resection age 16 consistent with PCOS. Uterine fibroid age 42. Presumed epithelial ovarian cancer age 50.	Mother of proband in (17)
P4^*c*^	**Type A insulin resistance with diabetes** *INSR* p.Phe409Val homozygous (rs121913142)	At age 8 with hirsutism. Menarche aged 12. At age 15, persistent hyperandrogenemia with polycystic ovarian morphology on pelvic ultrasound, prompting bilateral wedge resection. Left ovarian oophorectomy aged 19. Later GnRH analogue therapy (see Table 4).	19	N/A	>1000	Left ovary 2.43mL (excluding cyst). Numerous follicular cysts, stroma extensively luteinized with islands of thecal cells, consistent with PCOS. 9 × 9 × 8cm ovarian cystadenoma.	A5 in (22)
P5	**Rabson-Mendenhall syndrome** *INSR* p.Pro220Leu homozygous (rs749094324)	Treated from age 8 with leptin, high-dose insulin (900 units/day) and metformin. At age 19, amenorrheic with extensive hirsutism, hyperandrogenemia and bilaterally enlarged multicystic ovaries on pelvic ultrasound. Bilateral oophorectomy for hyperandrogenism. Testosterone undetectable after 8 months.	19	3688	648	Bilateral ovarian enlargement (left 60.5 mL, right 23.2 mL). Histology consistent with PCOS. Within this, a serous cystadenoma was identified.	RM-PaL in (23), female patient in (24), RMS-2 in (25)
P6	**Type A insulin resistance with diabetes** *INSR* p.Trp160*/p.Asn489Ser compound heterozygous (rs121913146, rs1135401742)	Insulin resistant diabetes from age 12 (>30,000 units/day). Menarche at age 15 with irregular menses thereafter. Hirsutism from age 23, clitoral index 150 mm^2^ (normal <35 mm^2^). Bilateral oophorectomy for massively enlarged ovaries and hyperandrogenism. Testosterone normalized after 3 months.	23	N/A	935	Bilateral ovarian enlargement (left 50 mL, right 36 mL). Numerous small dark cysts in both ovaries with abundant dense ovarian stroma, consistent with PCOS.	A1 in 4, 22, 26
P7	**Familial partial lipodystrophy with diabetes** Unknown genetic etiology	In early 20s with secondary amenorrhea, truncal and facial hirsutism, acne, acanthosis nigricans and hyperandrogenemia. Heterogeneous left ovarian mass on CT, bilateral oophorectomy age 27. Postoperatively, testosterone concentrations normalized, hirsutism persisted, no change in metabolic status.	27	212	332	Left ovary: numerous follicular cysts, stromal hyperthecosis, resembling PCOS. Within this, a steroid cell tumor was identified (Fig 4D-H)	-
P8	**Familial partial lipodystrophy with diabetes** *PPARG* p.Pro467Leu heterozygous (rs121909244)	In teens with generalized hirsutism and irregular menses after menarche aged 11. At age 17, ovarian biopsy consistent with PCOS. At age 28, COCP normalized menses and slowed hair growth for 6 months, and testosterone was undetectable. Hyperandrogenism recurred after discontinuation of therapy. Bilateral oophorectomy at age 29, following which testosterone undetectable.	29	2778	N/A	Histology consistent with PCOS.	A7 in (22)
P9	**Familial partial lipodystrophy with diabetes** *PPARG* p.Pro467Leu heterozygous (rs121909244)	Oligomenorrhoea and menorrhagia in 20s and 30s. Atypical polypoid hyperplasia within an endometrial polyp prompted total abdominal hysterectomy age 45. Presented age 48 with hirsutism, temporal alopecia, increased muscle bulk, voice change, acanthosis nigricans. Hyperandrogenemia with a left ovarian tumor. Symptoms regressed and testosterone normalized after bilateral salpingo-oophorectomy.	48	N/A	1240	144mL left ovary containing moderately differentiated Sertoli-Leydig cell tumor (Meyer’s type 2).	(27)

Abbreviations: COCP, combination oral contraceptive pill; TT, total testosterone; PCOS, polycystic ovary syndrome. N/A: data unavailable.

^*a*^Age corresponding to ovarian histology.

^*b*^Measured prior to oophorectomy or ovarian biopsy. Insulin was measured in the fasting state.

^*c*^Also reported in Table 4. Normal ovarian volume 5 mL.

### Utility of Gonadotropin Receptor Agonists in Disparate Etiologies of Severe Insulin Resistance

We previously reported that long-acting GnRH analogue therapy can lower testosterone levels and ameliorate hyperandrogenism in patients with primary SIR resulting from insulin receptor autoantibodies (P13; (30)). We now describe 6 patients (including P13) with SIR (2 with type B IR, 2 with pathogenic *INSR* variants, 1 with a pathogenic variant in *TBC1D4*, and 1 with acquired partial lipodystrophy associated with juvenile dermatomyositis) who received GnRH analogue therapy for management of hyperandrogenism (Table 4). One patient (P4) also underwent oophorectomy (Table 3). Four of the patients were postpubertal, 1 had primary amenorrhea and 1 was postmenopausal. All 6 patients experienced amelioration in hyperandrogenic symptoms and a reduction in TT to normal or near-normal levels in the absence of any change in their insulin sensitivity or glycemia control. In 5 patients, oophorectomy was avoided; 4 of these women were premenopausal with a desire for fertility preservation (P10-13), and 1 was post-menopausal but at high operative risk due to comorbidities (P15). One patient (P4), having previously undergone unilateral oophorectomy, underwent completion oophorectomy after GnRH analogue therapy further improved her symptoms.

**Table 4.** Biochemical response to gonadotrophin-releasing hormone analogues in primary severe insulin resistance

Patient	Clinical & genetic diagnosis	Clinical presentation	Age^*a*^, y	Before treatment					After treatment				Ref
				Insulin, pmol/L	TT, ng/dL	LH, U/L	FSH, U/L	GnRH analogue	Time^*b*^	TT, ng/dL	LH, U/L	FSH, U/L	
P10	**Insulin resistance with diabetes** *TBC1D4* 13:75324235 A>C splice site donor heterozygous (rs201722427)	Hair growth on chin, back, and chest age 12. Diabetes age 14, with worsening hirsutism, cystic acne (face and chest) and clitoromegaly (clitoral length 4 cm, index 42 mm^2^, normal <35 mm^2^). No improvement after metformin. 22.5 mg intramuscular leuprolide acetate administered age 15, after which testosterone levels decreased. No change in HbA1c after 6 months (insulin was not measured). Lost to follow up.	15	1051	334	3.6	2.9	Leuprorelin	16 days	100	2.3	1.7	
P11	**Rabson–Mendenhall syndrome** *INSR* p.Ile146Met homozygous (rs121913159)	At age 16 with primary amenorrhea, clitoromegaly and facial hirsutism requiring shaving. Ferriman–Gallwey score 14, Tanner III breast development, Tanner IV pubic hair, clitoral index 105 mm^2^ (normal <35mm^2^). Multiple small ovarian follicles but no large cysts on pelvic ultrasound. 11.25mg leuprorelin acetate depot injections initiated 8-weekly with COCP. Reduction in testosterone and clitoral index over 4 months, without reported changes in mood, libido or shaving frequency. Insulin sensitivity did not change.	16	1320	980	11.6	8.8	Leuprorelin	4 months	60	1.2	2.1	
P4^*c*^	**Type A insulin resistance** *INSR* p.Phe409Val homozygous (rs121913142)	See Table 2 for history prior to oophorectomy. Persistent virilization, hirsutism, and amenorrhea aged 23 (4 years post unilateral oophorectomy). Daily subcutaneous injections of leuprolide initiated. Serum testosterone improved but remained elevated. Underwent completion oophorectomy 5 years after GnRH initiation.	23	NA	912	27.5	10.4	Leuprorelin	1 year	73–267	NA	NA	A5 in (22)
P12	**Type B insulin resistance**	At aged 29 with secondary amenorrhea and symptomatic diabetes due to INSR autoantibodies. Lean with prominent hirsutism and acne. Bilateral bulky ovaries, stromal hyperplasia and proliferating immature follicles on MRI. Leuprorelin commenced; testosterone reduced by 75% after 2 months, insulin requirements remained high. Systemic lupus erythematosus diagnosed. Immunosuppressive therapy initiated with rapid improvement in glycemic control. Serum testosterone concentration normal after 24 months.	30	4749	1562	4	2	Leuprorelin	2 months	432	NA	NA	
P13	**Type B insulin resistance**	At age 29 with hyperinsulinemia and testosterone in the adult male range with INSR autoantibodies. Spontaneous remission of autoantibody with resolution of hyperandrogenemia. Autoantibody recurred 2 years later, manifesting as hyperglycemia, worsening acanthosis, voice changes, and increased shaving. Treatment with leuprorelin led to normalization of serum testosterone, despite persistent extreme insulin resistance, with decreased frequency of shaving, improved acne, softer voice, and better mood.	32	1715	778	7.8	5.5	Leuprorelin	2 months	33.7	0.4	1.8	(30)
P14	**Acquired partial lipodystrophy** (in childhood) with juvenile dermatomyositis	Diagnosed with PCOS in 30s (hirsutism and oligomenorrhoea), treated with cyproterone acetate. Hirsutism returned after cyproterone discontinued at age 51. MRI showed single ovarian cysts bilaterally (22mm and 19mm diameter). Medical comorbidities precluded oophorectomy therefore goserelin commenced (3.6 mg monthly). Facial hirsutism improved and testosterone levels normalized after 3 months. After 6 months, goserelin stopped, hirsutism returned, and serum testosterone concentration rose above normal. Goserelin restarted, and testosterone remained suppressed after 18 months.	53	300	444	21	35.2	Goserelin	3 months	14.4	4.8	19.3	

Abbreviations: COCP, combination oral contraceptive pill. FSH, follicle-stimulating hormone; INSR, insulin receptor; LH, luteinizing hormone; TT, total testosterone. N/A: data unavailable.

^*a*^Age at start of therapy.

^*b*^Time (in specified units) since onset of GnRH analogue therapy at re-evaluation.

^*c*^Also reported in Table 3. Insulin was measured in the fasting state.

## Discussion

There is compelling evidence that IR, with consequent hyperinsulinemia, drives hyperandrogenism and ovarian dysfunction. Adult-onset insulin receptor blockade by autoantibodies (type B IR) leads to acute, often severe hyperandrogenism and ovarian enlargement which reverses when antibodies, and hence IR, are cleared or remit (31, 32). Severe hyperandrogenism in several types of congenital SIR has also been described (16, 22, 33, 34). Here, we offer comprehensive assessment of androgen excess in primary SIR and confirm that hyperandrogenemia is common in female patients with SIR (34% of those studied had TT levels above the adult female reference range) and that it can be extreme, sometimes exceeding the adult male range.

Hyperandrogenemia occurs irrespective of IR etiology, being seen in insulin signaling disorders and lipodystrophy alike, and ovarian histopathology is indistinguishable from PCOS in each of these groups. Of biochemical note, higher TT concentrations in the insulin signaling subgroup did not correspond to higher FT concentrations, in keeping with a positive correlation of SHBG with an index of IR in this group, quite distinct from the *inverse* relationship between IR and SHBG seen in lipodystrophy or common forms of IR. This agrees with our previous finding that preserved or increased SHBG concentration in SIR is a discriminating marker of insulin receptor dysfunction (35). Although detailed evaluation of clinical hyperandrogenism in our cohort was beyond the scope of this study, our findings support a causal relationship between IR/hyperinsulinemia and PCOS, with ovarian hyperandrogenism resulting from excessive insulin action on the ovary. We did not find evidence for elevated circulating LH/FSH ratio among pubertal and postpubertal individuals, despite this being a recognized feature of PCOS. It is noteworthy that not all subjects with IR syndromes had elevated testosterone, and the correlation between serum insulin and testosterone was not strong (r_s_ = 0.211), suggesting that ovarian sensitivity to insulin may vary considerably among individuals.

“Insulin resistance” is usually defined in terms of attenuated ability of insulin to stimulate glucose uptake (36, 37). Until beta cell failure occurs, this is compensated for by hyperinsulinemia, with plasma insulin concentration raised by 1 or 2 orders of magnitude in severe cases. “Insulin resistance” does not apply equally to all of insulin’s actions, however, as evidenced by differences between simple insulin deficiency and IR. Some consequences of IR, including acanthosis nigricans and ovarian enlargement, require hyperinsulinemia, and presumably reflect increased insulin action (38-40). The ovary may thus be viewed as an “innocent bystander” in IR, suffering adverse trophic actions of the high insulin concentration required to maintain euglycemia. This is supported by hyperandrogenism and PCOS described in patients with insulinoma (41, 42), and in hyperinsulinemic patients with glycogen storage disease (43), and by increased prevalence of hyperandrogenism and PCOS-like ovarian appearances in patients with type 1 diabetes, attributed to supraphysiological exogenous insulin in peripheral circulation (44, 45).

Several mechanisms have been invoked to explain the mixed profile of insulin resistant and insulin sensitive phenomena seen in “insulin resistance.” One possibility is that different arms of the insulin signaling pathway are differentially affected by IR. For example, it has been suggested based on studies of muscle insulin signaling in PCOS that “metabolic” insulin signaling through the PI3K pathway is selectively impaired, leaving “mitogenic” signaling intact (46). Another model holds that high concentrations of insulin activate mitogenic insulin-like growth factor 1 (IGF1) receptors, which may be compounded by alterations in IGF binding proteins in IR (47).

If a selective defect in metabolic actions of insulin were critical to pathogenesis of IR-associated ovarian phenotypes, then defects in the insulin receptor, affecting all insulin signaling equally, would be associated with a less severe ovarian phenotype than defects in the PI3K/AKT2 arm of the signaling pathway. This was not observed. The ovarian phenotype we describe in an infant with Donohue syndrome, characterized by minimal residual insulin receptor function, suggests moreover that ovarian effects of extreme hyperinsulinemia are not mediated through the insulin receptor. These findings favor the hypothesis that insulin drives hyperandrogenism and PCOS via action on ovarian IGF1 receptors.

In keeping with this, the ovarian histopathology we describe in diverse forms of SIR demonstrates commonality in appearances irrespective of age/pubertal stage and monogenic defect. All 4 patients with pathogenic variants in *INSR* showed features consistent with PCOS, including numerous follicular cysts within an extensively luteinized stroma associated with dense stromal proliferation. Other features, such as larger cysts or massive ovarian enlargement, were more variable. Features of PCOS were similarly observed in 3 patients with partial lipodystrophy, including 1 patient with digenic IR (P2) and 1 patient with heterozygous pathogenic variants in *PPARG* (P8).

Most published series suggest that a testosterone concentration of around 150 ng/dL (~5 nmol/L) is a reasonable trigger for screening for virilizing tumors (48, 49). Serum testosterone concentration in excess of this level was observed in 13% of women/girls in our cohort. Our case series demonstrates that SIR is sufficient to induce hyperandrogenemia sometimes in excess of this threshold, which is usually not of tumoral origin. On the other hand, 5 patients with SIR of different etiologies developed ovarian tumors associated with virilization. Added to reports of neoplasia arising in the context of sustained severe hyperinsulinemia, ovarian hyperthecosis and SIR (13), this suggests that IR-related hyperandrogenemia may not be entirely benign in the long term. We suggest that long-term hyperinsulinemia may increase risk of virilizing ovarian tumors. Screening strategies for autonomous tumors need to be evaluated, possibly using intermittent GnRH suppression testing to look for evidence of autonomous androgen secretion.

Finally, we report the treatment of 6 patients with hyperandrogenism due to SIR with gonadotropin suppression, adding to existing literature (30). All exhibited testosterone lowering into the normal female range or frank suppression, and experienced marked symptomatic improvement, supporting a requirement of pulsatile gonadotropins for the adverse actions of hyperinsulinemia to cause ovarian hyperandrogenism. This is in keeping with the synergic action of insulin and luteinizing hormone on ovarian thecal cells in vitro to drive thecal cell hyperplasia and androgen synthesis (50-52). Suppression of gonadotropins with longer-acting GnRH analogues may thus be a useful therapeutic option for hyperandrogenic patients in a broad range of SIR subphenotypes, and warrants further study in several settings. In infants with Donohue syndrome and marked ovarian enlargement, GnRH analogues may be considered to prevent ovarian enlargement that in some cases is severe, while in young women with extreme IR and hyperandrogenism they may be used in combination with hormone replacement in a “block and replace” strategy when future fertility is desired. This may be an undesirable strategy in dyslipidemia, for example due to lipodystrophy, where estrogens may exacerbate hypertriglyceridemia, however (53). In postmenopausal women, GnRH agonist therapy may allow oophorectomy to be avoided when surgical risk is high, as reported in idiopathic post-menopausal hyperthecosis (54-56). A note of caution is sounded by a recent report of progressive ovarian enlargement in a woman with SIR treated with long-term GnRH analogue therapy despite effective treatment of hyperandrogenemia. This was attributed to gonadotropin-independent mitogenic actions of insulin on granulosa cells (57).

The retrospective, historical nature of our data is a limitation of this study. In particular, we report TT concentrations determined using various platforms in different referring centers over several decades. Nevertheless, most of these were immunoassay-based measurements and thus broadly comparable. A small minority were determined by mass spectrometry; given the increased specificity of this technique, we would anticipate even higher levels of hyperandrogenemia had they been measured by immunoassay instead. FT was not measured directly, but estimated from TT and SHBG, assuming normal albumin, using conventional methods. Medication (including insulin) history was recorded in only a subset of individuals and not incorporated into analyses. Finally, whilst we did not programmatically exclude potentially confounding pathophysiology (such as dysthyroidism, hyperprolactinemia, or liver/adrenal disease) in all study participants, this was undertaken in referring centers prior to referral. Overall, the unique size and rarity of this patient cohort allows important, generalizable lessons to be drawn in spite of these drawbacks.

IR is reported in up to 70% of cases of “common” PCOS (58, 59) but the direction of causality is debated. Some evidence suggests that primary hyperandrogenemia may cause IR. For example, nonclassical congenital adrenal hyperplasia (prior to glucocorticoid replacement) is associated with decreased insulin sensitivity (60) as is androgen use by healthy women (61, 62). The effects of pharmacological androgen reduction or androgen receptor antagonists on insulin sensitivity have been less consistent (63); however, Mendelian randomization in a population-based study has supported a causal link between hyperandrogenemia and both PCOS and type 2 diabetes (64). In keeping with these human observations, rodents exposed to exogenous androgens exhibit many features of PCOS (65). More recently, adipose-derived androgens, which are increased in PCOS, have been proposed to drive metabolic abnormalities in PCOS (66). Our data do not provide additional support for this hypothesis, as androgen reduction after GnRH analogue administration or oophorectomy did not discernibly alter insulin sensitivity in severe, primary IR. This does not exclude a bidirectional relationship between hyperandrogenemia and IR, although we suggest that the effect of IR on androgen secretion is likely far greater than the effect of hyperandrogenemia on insulin sensitivity, based on our findings.

## Data Availability

Restrictions apply to the availability of some or all data generated or analyzed during this study to preserve patient confidentiality or because they were used under license. The corresponding author will on request detail the restrictions and any conditions under which access to some data may be provided.

## Abbreviations

BMI: body mass index
COCP: combination oral contraceptive pill
DS: Donohue syndrome
FSH: follicle-stimulating hormone
FT: free testosterone
GnRH: gonadotropin-releasing hormone
HPG: hypothalamic–pituitary–gonadal
INSR: insulin receptor
IQR: interquartile range
IR: insulin resistance
LH: luteinizing hormone
PCOS: polycystic ovary syndrome
RMS: Rabson-Mendenhall syndrome
SHBG: sex hormone–binding globulin
SIR: severe insulin resistance
TT: total testosterone

## Additional Information

***Disclosures*:** The authors have no conflicts of interest relevant to this article to disclose.
